# Sandfly (Diptera: Psychodidae: Phlebotominae) species diversity in an urban area of the municipality of Tapachula, Chiapas, Mexico

**DOI:** 10.1590/0074-02760140351

**Published:** 2015-02

**Authors:** Oscar Fernando Mikery Pacheco, Julio Cesar Rojas León, Eduardo Alfonso Rebollar-Téllez, Alfredo Castillo Vera

**Affiliations:** 1 Ecología de Artrópodos y Manejo de Plagas, El Colegio de la Frontera Sur, Tapachula, Chiapas, México; 2 Departamento de Zoología de Invertebrados, Facultad de Ciencias Biológicas, San Nicolás de los Garza, Nuevo León, México

**Keywords:** sandflies, urban area, Mexico, urbanisation

## Abstract

Monitoring phlebotomine sandflies in urban areas is key for epidemiological studies in susceptible populations. This paper describes sandfly fauna that were present in an urban area of the municipality of Tapachula, Chiapas, Mexico, and were captured with Shannon and CDC light traps. During February and March of 2014, 1,442 sandflies were captured, specifically Lutzomyia cruciata (Coquillet) (98.8%), Lutzomyia cayennensis cayennensis (Floch and Abonnenc) (0.8%), Lutzomyia chiapanensis (Dampf) (0.3%) and Lutzomyia atulapai (De León) (0.1%). Lu. cruciata was the most abundant and the most frequently trapped species. This is the first record of its remarkable ability to adapt to urban green areas. The three other species trapped represent new records of geographic distribution for the study region. These results indicate the need to establish measures for reducing both human contact with this vector and the risk of possible sites of infection.

Chiapas is one of the states of the Mexican Republic with high incidence of skin and visceral leishmaniasis (Sanchez-Tejeda et al. 2001, Becker et al. 2005). The main risk factors for contracting this disease are urbanisation of ecosystems, poor host immunological status and lack of effective treatment (Dujardin 2006). Environmental changes, which can also be the product of urbanisation, increase human exposure to phlebotomine sandflies as a consequence of the alteration in range and density of the vectors (Dujardin 2006). This paper describes, for the first time, the composition of sandfly fauna present in a green area property of the Technological Institute of Tapachula (ITT) located in the urban zone (160 m above sea level) of Tapachula, Chiapas, Mexico (Figure). This area is characterised by the presence of a large variety of tree and bush species, among which Mangifera indica, *Theobroma cacao* and *Coffea* sp. predominate. The availability of vertebrates, which represent sources of blood for the sandfly and possible reservoirs of the disease (Young & Arias 1992, Rotureau 2006), is unknown. Seven miniature light traps of the CDC type and a Shannon trap with two or three people (protected from the bites of bloodsucking insects) inside the trap served to attract the sandflies. Traps of both types were placed within the area for 14 inconsecutive nights (06:30 pm-08:30 pm) during the dry season (February-March 2014). The captured insects were deposited in containers with 70% alcohol. To identify the material, permanent preparations were made in Euparal^(r)^ resin (BioQuip Products, USA) using the method described by Ibáñez-Bernal (2005a). The keys of Ibáñez-Bernal (1999, 2003, 2005a, b) were used for morphological identification of the captured samples.

**Figure:**
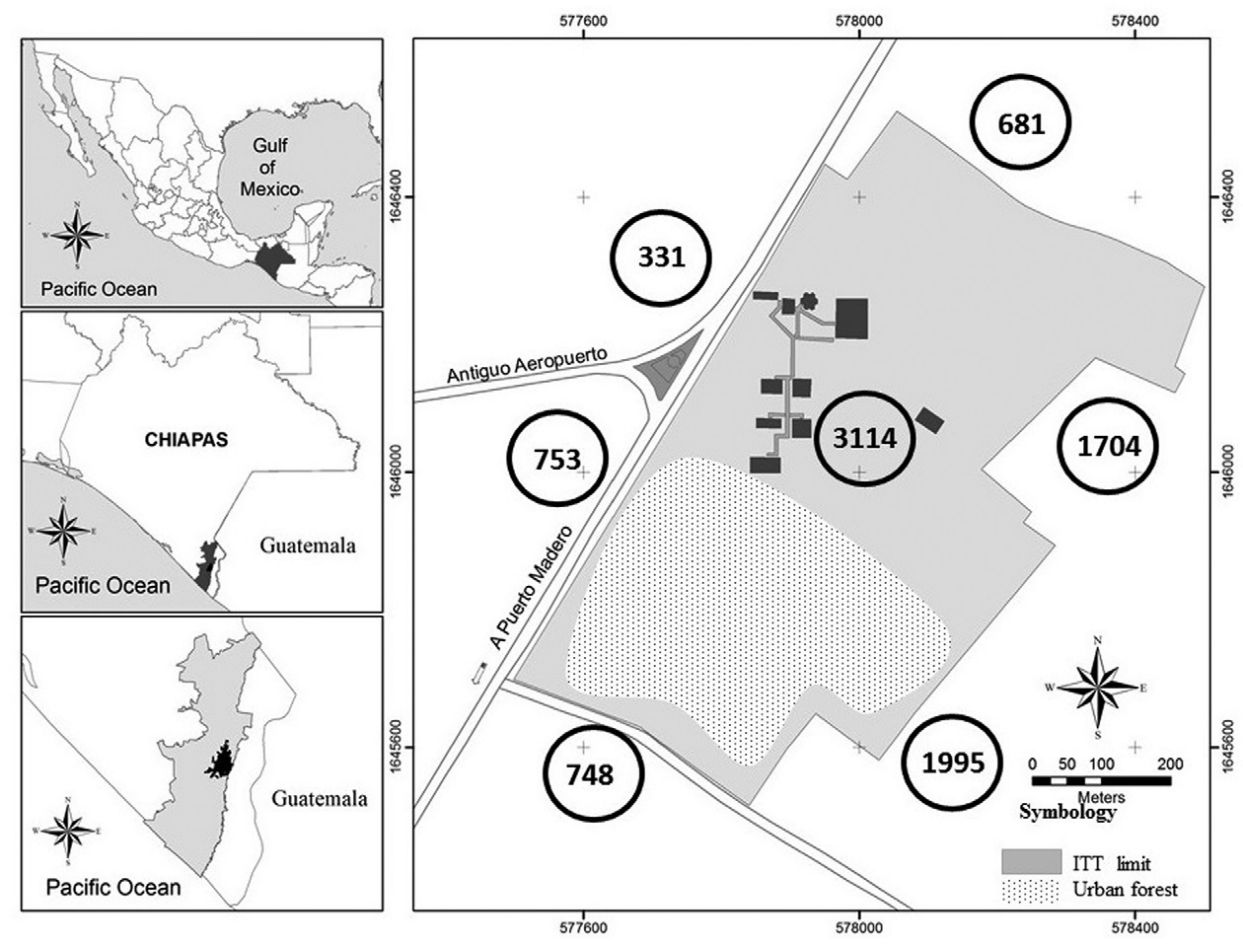
Location of the sample site in the municipality of Tapachula, Chiapas, Mexico. Numbers inside the circle indicate number of inhabitants, the dotted area indicates the sampled forest and black rectangles indicate the classrooms. ITT: Technological Institute of Tapachula.

A total of 1,442 phlebotomine sandflies were captured in the study area using the two types of traps (CDC = 74; Shannon = 1,368). The species identified were *Lutzomyia cruciata* (98.8%), *Lutzomyia cayennensis cayennensis* (0.8%), *Lutzomyia chiapanensis* (0.3%) and *Lutzomyia atulapai* (0.1%) (Table). This paper presents the first description of the composition of the sandfly population within an urban area of this region of Mexico. Registers of the presence of *Lu. cruciata* in the coffee-producing region of Soconusco date from 1941 (Ibáñez-Bernal 1999), the oldest geographic record of the four captured species. This, however, is the first time the species has been trapped in an urban area. Both types of traps captured mostly specimens of *Lu*. *cruciata*, confirming its anthropophilic habits (Rebollar-Téllez et al. 1996), although that species was more predominant in the Shannon trap. The high catches of this species reflect its high capacity to adapt to this type of habitat. Maintenance of this wild population must have required a relatively nearby food source, considering the limited dispersion capacity of haematophagous adults (< 100 m). The vertebrates associated with the habitat certainly provide ideal conditions for its development, inactive phase and reproduction (Wasserberg et al. 2003, Salomón et al. 2008). The approximately 9,326 people who live near the study area [INEGI (inegi.org.mx/), DGEST/ITT (2013)], however, should not be ruled out as a source of food for *Lu. cruciata*. This study is the first geographic record of the species *Lu. c. cayennensis*, *Lu. chiapanensis* and *Lu. atulapai* in this area. Records of these species in Mexico are rare, especially in Chiapas. In 1959, *Lu. c. cayennensis* was collected from a hole in a tree in the municipality of Acacoyahua, Chiapas, co-existing with bats and reptiles (Vargas & Díaz-Nájera 1959). *Lu. chiapanensis* was collected in 1938 in the municipality of Chiapa de Corzo (Ibáñez-Bernal 2003) and, in the same year, *Lu. atulapai* was trapped in the municipality of Tuxtla Gutiérrez, Chiapas (Ibáñez-Bernal 2001). Previous records of the presence of these species in Chiapas are very old, which limits the possible conclusions, although there may be geographic records in new areas as a consequence of highly modified anthropology. A new geographic record for *Lu. cruciata* would constitute high risk of human contact with the vector and indicate possible foci of infection because of the medical importance of this species (Pech-May et al. 2010) and because the conditions in this region favour population increases during the dry season (Pérez et al. 2014).

**TABLE:** Phlebotomine species captured in an urban area of the municipality of Tapachula, Chiapas, Mexico

	Shannon			CDC		
Species	♀	♂		♀	♂	Total
*Lutzomyia cruciata*	1,368	0		28	28	1,424
*Lutzomyia cayennensis cayennensis*	0	0		6	6	12
*Lutzomyia chiapanensis*	0	0		4	0	4
*Lutzomyia atulapai*	0	0		0	2	2

Association of phlebotomine sandflies with patches of vegetation in urban areas has been documented in other parts of the world with endemic leishmaniasis (Salomón et al. 2008, Cerino et al. 2009). Tapachula has a population of approximately 350 thousand inhabitants [INEGI (inegi.org.mx/)], most of whom are people from other parts of Chiapas, other states within Mexico and most of them are from Central America. In some of these places, leishmaniasis is endemic. Modifications in the landscape, together with peridomestic transmission and domestication of the vector, promote epidemic outbreaks (Salomón et al. 2008). Currently, there are no clinical cases of the disease recorded in this city, although *Lu. cruciata* is a species that has been confirmed as vector of leishmaniasis in Mexico (Pech-May et al. 2010). The combination of the possible immigration of infected humans to urban environments and the abundance of vector species would threaten the population with outbreaks of leishmaniasis. The environmental conditions of urban zones are adverse compared with those in secondary vegetation patches, although the latter may be comparable to those observed in peridomestic habitats (Salomón et al. 2008). In addition to the high abundance of *Lu. cruciata*, the other species captured, although in low numbers, have the ability to feed on mammals or other domestic animals (Dampf 1947, Cochero et al. 2007) and thus also represent a risk of leishmaniasis transmission through interaction between possible zoonotic and anthropozoonotic cycles that may arise (Chaves & Añez 2004).

This paper is the first description of sandfly population composition within an urban area in this region of Mexico and the findings are the first record of *Lu. cruciata* in this type of habitat, suggesting that it is highly capable of adapting to urbanised environments. This finding makes it necessary to explore other sites with the same conditions and to monitor population fluctuations with the combined objective of establishing measures to reduce human contact with the vector and to reduce the risk of possible foci of infection.
